# Supplementary material for: Genetic Susceptibility and Genetic Variant-Diet Interactions in Diabetic Retinopathy: A Cross-Sectional Case–Control Study
Source: Nutrients. 2025 Sep 17;17(18):2983. doi: 10.3390/nu17182983 (PMC12472948; doi:10.3390/nu17182983)
Supplement: Supplementary file 1 [file nutrients-17-02983-s001.zip › nutrients-3858370-supplementary.pdf]

Table S1. Characteristics of SNP-SNP interactions and their influence on diabetic retinopathy risk analyzed through generalized multifactor dimensionality reduction (GMDR) in a Korean adult population

GMDR	No Adjustment				Adjusted for age, gender, residence area, diabetic duration BMI, hypertension, eGFR, serum triglyceride, eGFR, smoking, alcohol intake, and physical activity, and medications.			
Model	TRBA[a]	TEBA[b]	P value	CVC[c]	TRBA	TEBA	P value	CVC
*ABCA4*_rs17110929	0.7169	0.7226	10(0.0010)	10/10	0.6807	0.6836	10(0.0010)	10/10
Model 1 plus *MMP2*_rs2576531	0.7543	0.7423	10(0.0010)	10/10	0.7136	0.7021	10(0.0010)	10/10
Model 2 plus *FOXP1*_rs557869288	0.7863	0.7802	10(0.0010)	10/10	0.7403	0.7327	10(0.0010)	10/10
Model 3 plus *IGSF21*_rs573262	0.7971	0.7817	10(0.0010)	9/10	0.7491	0.7275	10(0.0010)	6/10
Model 2 plus *HLA-DQB1*_rs9274247 *RBFOX1*_rs733616 IGSF21_rs573262	0.8091	0.6935	10(0.0010)	5/10	0.7603	0.6774	10(0.0010)	4/10
Model 2 plus *MRPS33*_rs1533933 *HLA-DQB1*_rs9274247 *RBFOX1*_rs733616 *GABRB3*_rs72712070	0.8383	0.6060	10(0.0010)	5/10	0.7855	0.6622	10(0.0010)	10/10
Model 6 plus *IGSF21*_rs573262	0.8689	0.5930	10(0.0010)	9/10	0.8079	0.5869	10(0.0010)	7/10
Model 7 plus *FOXP1*_rs557869288	0.8861	0.6087	10(0.0010)	7/10	0.8281	0.5856	10(0.0010)	9/10
Model 8 plus *DRD2*_rs4936270	0.8991	0.6140	10(0.0010)	10/10	0.8428	0.6154	10(0.0010)	10/10
Model 9 plus *TRPV3*_ rs56899958	0.9068	0.5808	10(0.0010)	10/10	0.8519	0.5908	10(0.0010)	10/10

SNP, single-nucleotide polymorphism; eGFR, estimated glomerular filtration rate; [a]TRBA, trained balanced accuracy; [b]TEBA, test balance accuracy; [c]CVC, cross-validation consistency.

Table S2. Stratified analysis of polygenic risk score (PRS) and DM-DR by Diabetes Duration

	Low-PRS	Medium-PRS	High-PRS
No exclusion (DM-NR: n=4,873; DM-DR: n=165)	1	8.87 (5.79 – 13.60)	50.39(30.84 – 82.33)
DM duration >20 yrs (DM-NR: 4,873; DM-DR: n=112)	1	7.79(4.79 – 12.67)	37.09(21.02 – 65.43)
DM duration >10 yrs (DM-NR: n=4,408; DM-DR: n=54)	1	7.26(3.67 – 14.34)	33.77(15.55 – 73.33)
P value for Q		0.992	0.609
I^2		0	0

Covariates: age, gender, body mass index, smoking, alcohol intake, physical activity, hypertension, serum triglyceride, and medication. PRS, polygenic risk scores; ND, non-diabetics; DM-NR, diabetes without retinopathy; DM-DR, diabetes with retinopathy

Table S3. Adjusted odds ratios and 95% confidence intervals for diabetic retinopathy by polygenetic risk scores of the best model (PRS-3SNP) without covariate adjustments according to lifestyles.

	Low-PRS (n=3,880)	Medium-PRS (n=1,038)	High-PRS (n=192)	Interaction of PRS-3SNP in DM-NR vs DM-DR
Low fruit	1	6.756 (3.069-14.87)	59.66 (26.80-132.83)	0.0084
High fruit	1	8.953 (5.489-14.61)	48.17 (27.61-84.01)	
Low fast foods	1	7.474 (3.656-15.28)	36.644 16.074 83.539	<0.0001
High fast foods	1	4.700 (1.168-18.91)	19.583 3.376 113.609	
Low coffee	1	8.379 (4.421-15.88)	43.14 (20.37-91.37)	<0.0001
High coffee	1	8.339 (4.804-14.48)	55.14 (30.53-99.58)	
Low alcohol	1	8.263 (5.008-13.63)	42.107 (16.25-109.14)	<0.0001
High alcohol	1	11.274 (5.190-24.49)	54.77 (31.99-93.78)	
Low eating speed	1	10.32 (5.222-20.38)	40.99 (18.78-89.47)	<0.0001
High eating speed	1	7.149 (4.238-12.06)	56.73 (32.47-99.10)	
Low exercise	1	6.956 (1.987-24.35)	32.98 (8.594-126.59)	<0.0001
High exercise	1	8.377 (5.367-13.07)	50.43 (30.33-83.85)	

Covariates: age, gender, diabetic duration BMI, hypertension, serum triglyceride, estimated glomerular filtration rate, smoking, alcohol intake, hypertension, serum triglyceride, and physical activity, and medications.

References: Fruit: <1 serving/day; fast foods: <1 serving/day; coffee: <0.5 cup/day; alcohol: <3 g/day; eating speed: 15 min/day; exercise: 30 min/day moderate intensity exercise.

Table S4. Adjusted odds ratios and 95% confidence intervals for diabetic retinopathy by low and high lifestyles according to polygenetic risk scores of the best model (PRS-3SNP)

	Low intake (reference)	High intake without adjusted covariates	High intake with adjusted covariates
Fruit			
Low-PRS	1	0.628 (0.300-1.317)	0.534 (0.229-1.246)
Medium-PRS	1	0.474 (0.271-0.830)	0.422 (0.173-0.954)
High-PRS	1	0.778 (0.413-1.466)	0.554 (0.117-2.628)
Fast foods			
Low-PRS	1	6.671 (0.882-50.47)	10.78 (3.653-31.808)
Medium-PRS	1	5.858 (2.200-15.60)	6.508 (2.183-19.400)
High-PRS	1	3.909 (0.903-16.93)	6.757 (0.805-28.495)
Coffee			
Low-PRS	1	3.722 (1.882-7.362)	4.986 (2.056-12.09)
Medium-PRS	1	3.740 (2.273-6.153)	3.128 (1.152-8.489)
High-PRS	1	2.912 (1.492-5.685)	5.145 (0.869-30.46)
Alcohol			
Low-PRS	1	10.39 (5.246-20.59)	8.263 (5.008-13.63)
Medium-PRS	1	6.214 (1.399-27.61)	5.825 (3.190-10.64)
High-PRS	1	3.636 (1.554-8.509)	1.586 (0.167-15.02)
Eating speed			
Low-PRS	1	1.495 (0.652-3.425)	1.276 (0.629-2.587)
Medium-PRS	1	1.063 (0.438-2.578)	1.180 (0.721-1.932)
High-PRS	1	1.759 (0.899-3.444)	3.936 (0.742-20.89)
Exercise			
Low-PRS	1	4.149 (1.358-12.68)	7.852 (2.76-22.31)
Medium-PRS	1	9.353 (4.243-20.62)	9.250 (3.210-26.65)
High-PRS	1	8.731 (3.515-21.69)	14.21 (1.987-101.65)

Covariates: age, gender, diabetic duration BMI, hypertension, serum triglyceride, estimated glomerular filtration rate, smoking, alcohol intake, hypertension, serum triglyceride, and physical activity, and medications.

Supplementary Figures

Figure S1. A flowchart of the selection of the participants.

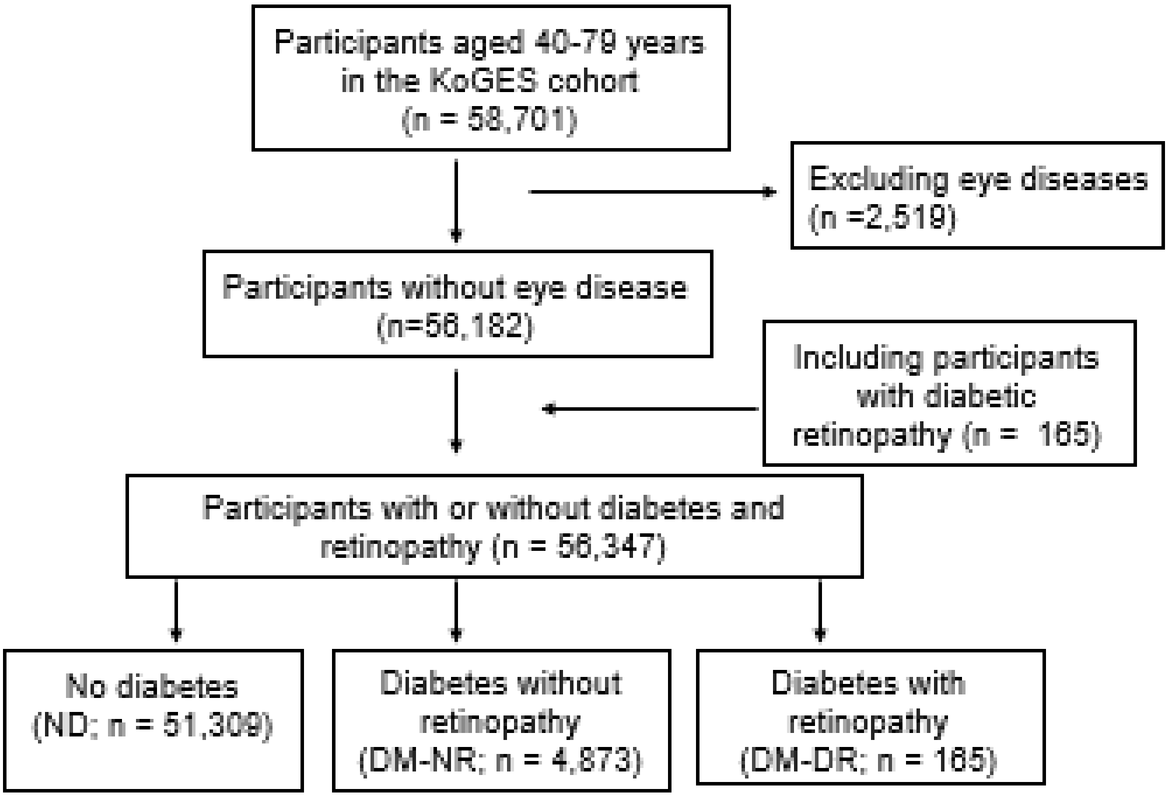

Figure S2. Examples of the fundus images

A. Normal

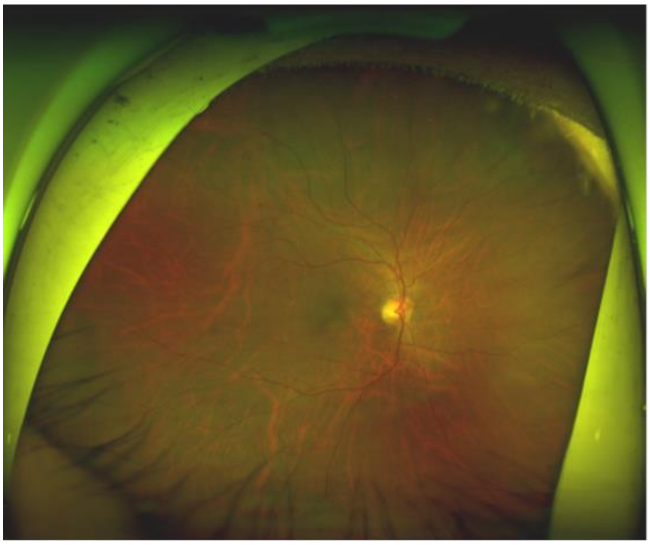

B. DM-DR (mild)

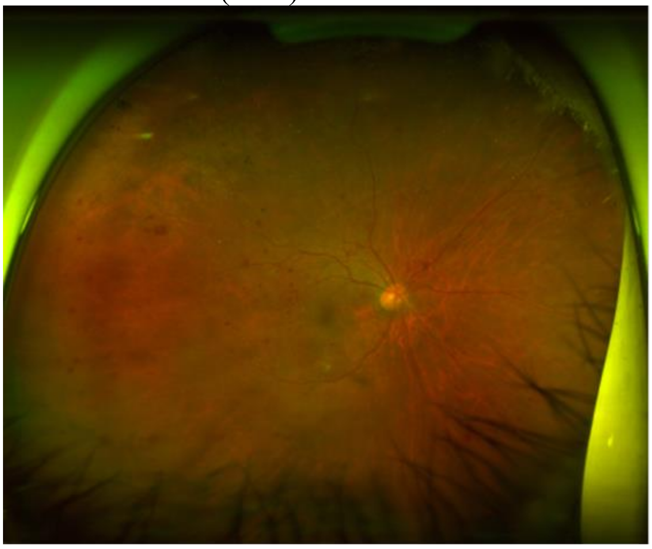

C. DM-DR (moderate)

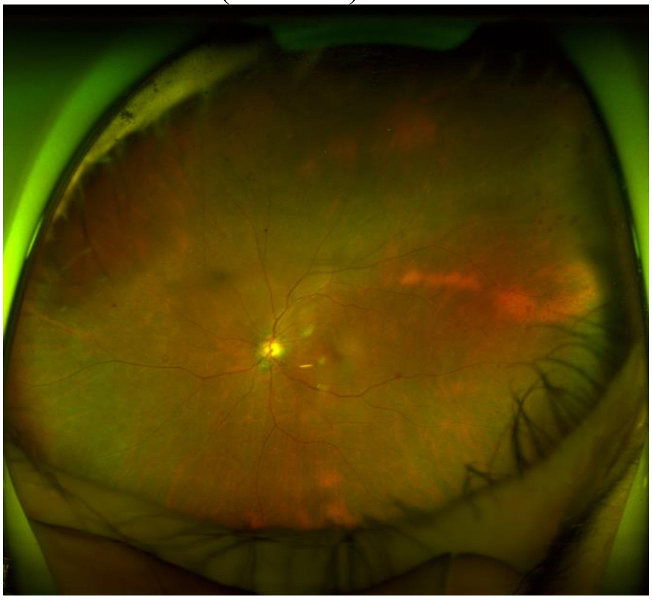

Fig. S3. The distribution of genetic variants related to diabetic retinopathy.

A. Manhattan plot of genetic variants related to diabetic retinopathy

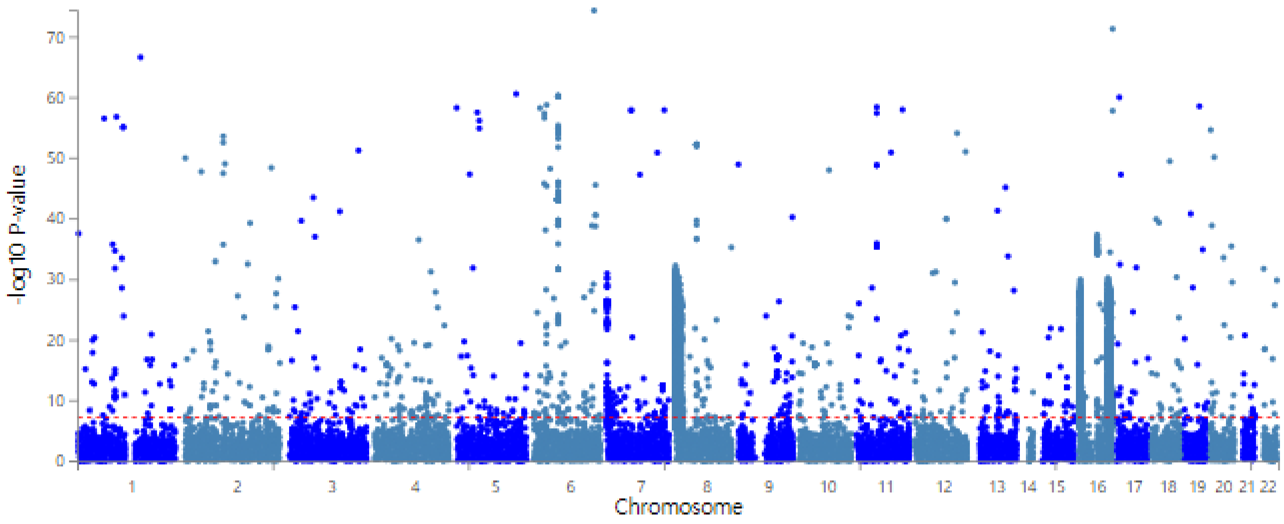

B. Q-Q plot of genetic variants related to diabetic retinopathy (λ=1.008).

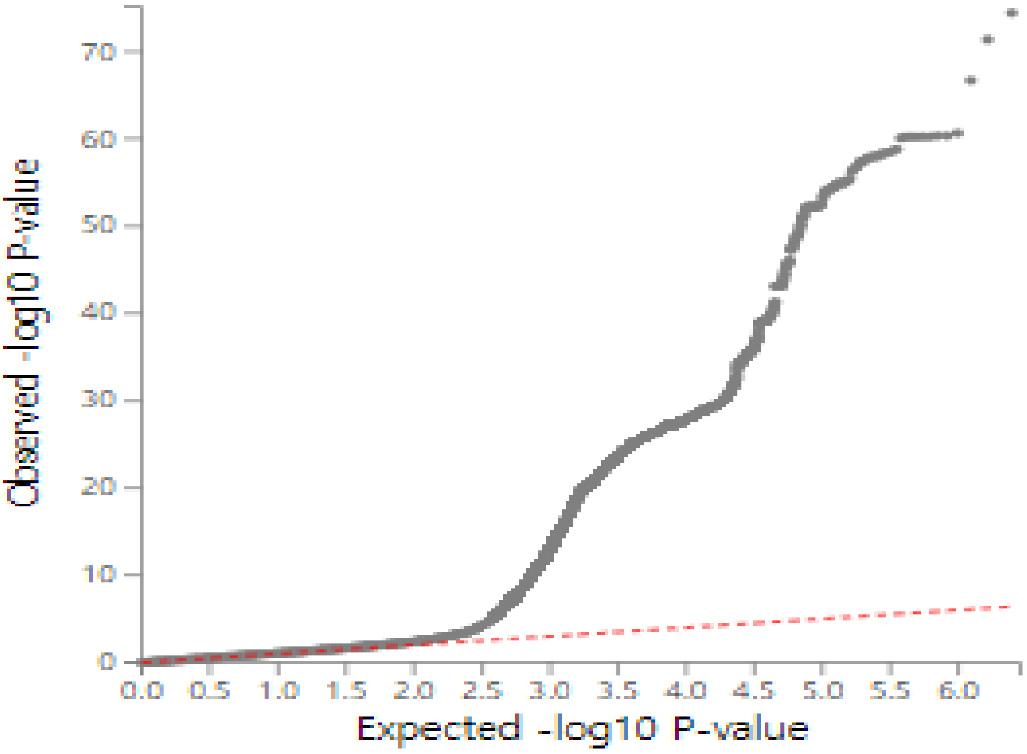

C. Histogram of the polygenic risk factor with 3 single-nucleotide polymorphisms (SNPs).

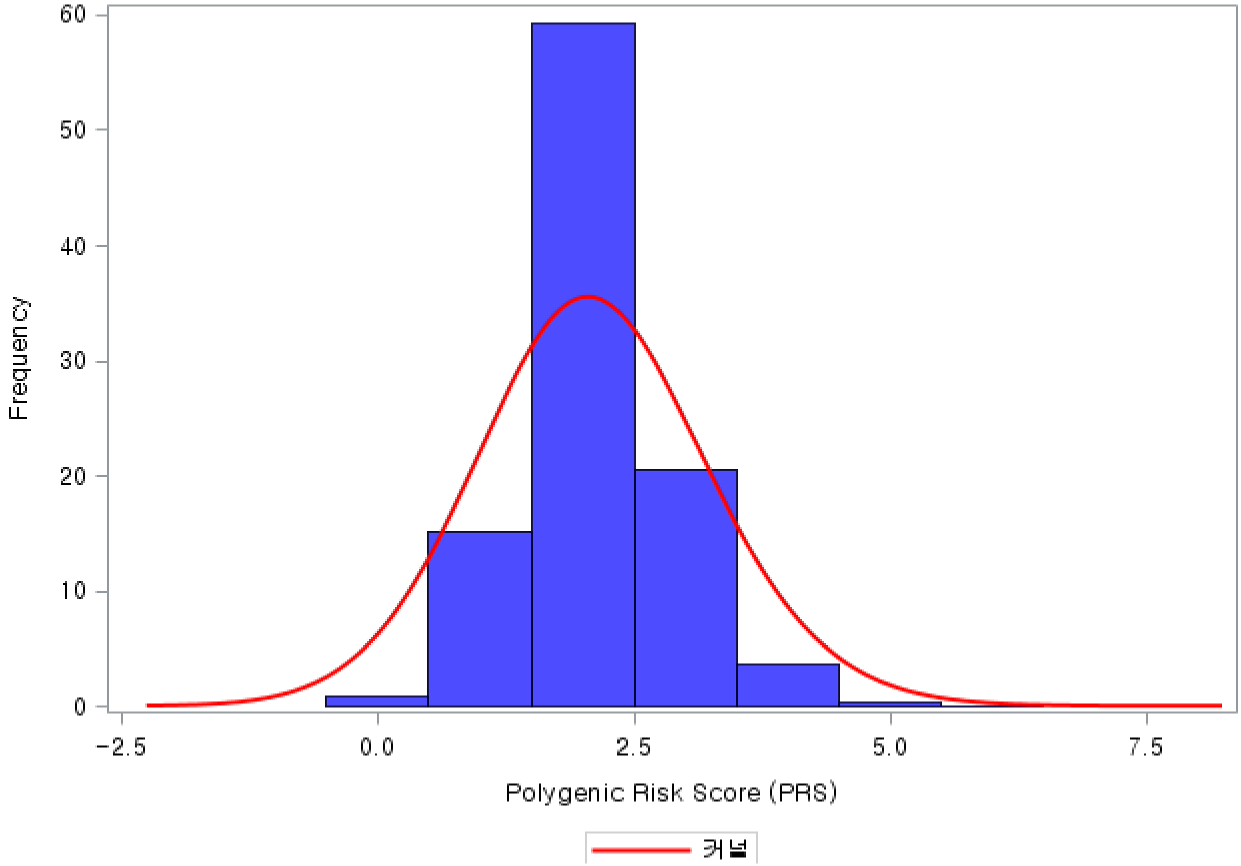

Fig. S4. Discriminative performance of the 3-SNP, 9-SNP, and 10-SNP polygenic risk score (PRS) models for predicting diabetic retinopathy (DM-DR)

A. ROC curve for 3-SNP PRS

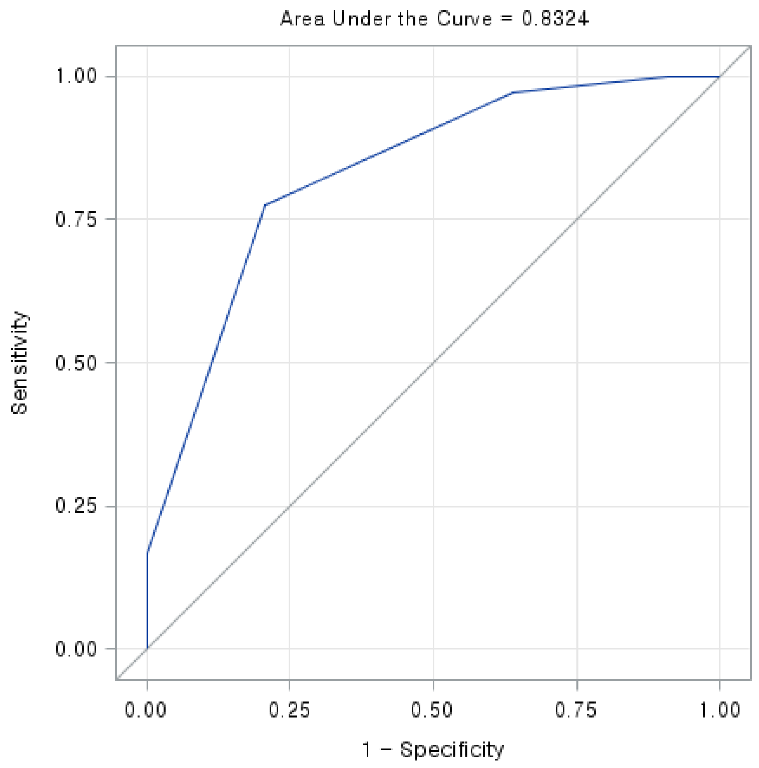

B. ROC curve for 9-SNP PRS

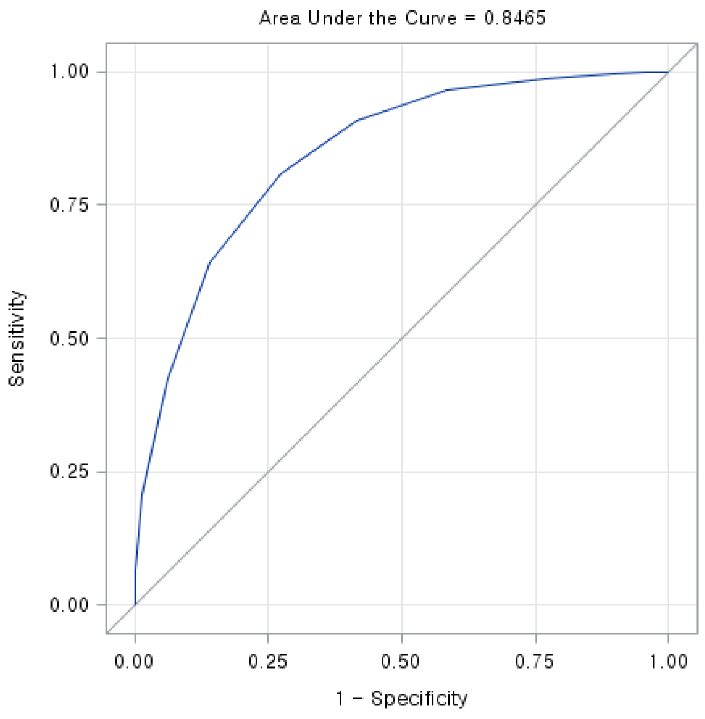

C. ROC curve for 10-SNP PRS

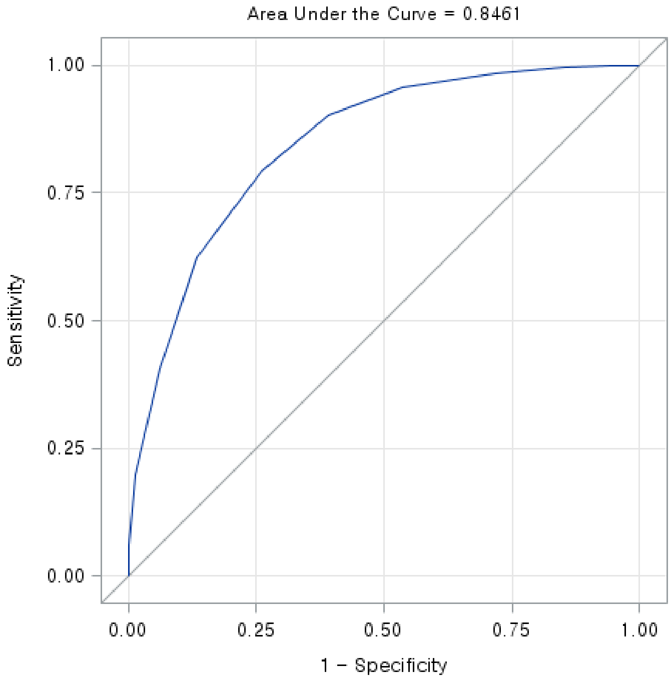

Method S1. Detailed methodologies for all genetic analyses (SNP selection, PRS construction, and pathway analysis)

2.5. Genotyping and quality control

Genomic DNA extracted from the blood samples of the Korean participants was processed using the Korean Chip (Affymetrix, Santa Clara, CA) at the Korea Centers for Disease Control and Prevention (KCDC) [1]. To ensure the reliability of the genotyping data, the accuracy of genotyping was assessed using the Bayesian robust linear model with the Mahalanobis distance classifier (BRLMM) genotyping algorithm [2]. SNPs were deemed acceptable if they met specific criteria, including a genotyping accuracy of ≥98%, heterozygosity of <30%, missing genotype call rate of <4%, Hardy-Weinberg equilibrium (HWE) with P>0.05, and no evidence of gender bias. Subsequently, KCDC provided the SNP data of the KoGES participants for the study.

2.6. Selection of SNPs for DM-DR association by GWAS

GWAS was performed between DM-NR and DM-DR groups, adjusting for age, gender, BMI, diabetes duration, medication use, hypertension, eGFR, serum triglycerides, alcohol consumption, smoking status, and physical activity [3]. Population stratification was assessed by calculating the genomic inflation factor (λ) from the GWAS test statistics and examining quantile-quantile (Q-Q) plots of the observed versus expected p-values. All participants were confirmed to be of Korean ancestry based on self-reported ethnicity. The genomic inflation factor was $\lambda = 1.05$, indicating minimal population stratification within the Korean cohort, which is consistent with the homogeneous ancestry of the study population.

SNPs with Hardy-Weinberg equilibrium P>0.05 and minor allele frequency <0.01 were excluded. Initially, 1048 genetic variants were selected at a genome-wide significance threshold of $P<5\times10^{-7}$, which represents a Bonferroni-corrected significance level ($\alpha = 0.05$) accounting for multiple testing

across the genome-wide SNP array between DM-NR and DM-DR. Subsequently, 282 unique gene names were identified from 884 of the 1,084 SNPs using g:Profiler (https://biit.cs.ut.ee/gprofiler/snpense). From 884 genetic variants, linkage disequilibrium-based pruning using R software ($r^2 < 0.8$) retained 103 independent SNPs. Twenty-six candidates were prioritized through a systematic literature review and Human Genome Epidemiology Navigator database, with 10 variants selected for epistatic analysis using the GeneMANIA prediction server (Table S1). Complete genetic variant selection, PRS construction, and pathway analysis methodologies are detailed in Supplementary Methods S1.

2.7. SNP-SNP Interaction Analysis and PRS Construction

SNP Selection and Initial Screening: Twenty-six candidate genetic variants associated with DM-DR were initially identified from 103 genetic variants through a systematic literature review and the Human Genome Epidemiology (HuGE) Navigator database. These variants were prioritized based on biological pathway relevance to DM-DR pathogenesis, reported disease associations, and functional significance. Ten genetic variants with the strongest predicted functional connectivity were selected using the GeneMANIA prediction server (https://genemania.org). **SNP-SNP Interaction Analysis:** Epistatic interactions among the ten selected SNPs were evaluated using Generalized Multifactor Dimensionality Reduction (GMDR), a non-parametric, model-free approach specifically designed to detect non-linear gene-gene interactions [4]. GMDR was selected over traditional logistic regression due to its superior capacity for identifying high-order epistatic effects. The analysis incorporated two covariate adjustment strategies: covariate set 1 (age, gender, BMI, diabetes duration) and covariate set 2 (covariate set 1 plus medication use for lipid-lowering, antihypertensive, hypoglycemic agents, hypertension, eGFR, serum triglycerides, alcohol consumption, smoking status, and physical activity) [3].

Model Development and Selection: Multiple PRS models incorporating 1 to 10 SNPs were

systematically constructed and evaluated. Model selection criteria required: (1) cross-validation consistency: 10/10, (2) P-value for testing balanced accuracy: <0.001, and (3) statistical significance in GMDR analysis with appropriate correction for multiple model comparisons. The final 3-SNP model was selected based on the genome-wide significance of individual variants, strongest epistatic effects, and clinical applicability considerations favoring parsimony. **Genetic variants and PRS calculation:** The final PRS model incorporated three SNPs identified through GMDR analysis including adenosine triphosphate (ATP)-binding cassette subfamily A member 4 (*ABCA4*)_rs17110929, *MMP2*_rs2576531, and *FOXP1*_rs557869288. Risk allele definitions were based on increased DM-DR association in our Korean cohort. PRS calculation was according to PRS = Σ(number of risk alleles across three SNPs) Where each SNP contributes 0, 1, or 2 risk alleles per individual, resulting in total scores ranging from 0-6. Low-risk individuals with PRS scores of 0-2 (n=3,880), medium-risk with scores of 3-4 (n=1,082), and high-risk individuals with scores of 5-6 (n=192). **Methodological rationale:** An unweighted count-based approach was employed [5] because: (1) GMDR analysis focuses on epistatic interactions rather than additive effects, (2) external effect size estimates for epistatic interactions are not available, and (3) this maintains clinical simplicity. This discovery-based approach identified variants within the same Korean cohort, avoiding reliance on external effect sizes from databases such as UK Biobank and DIAGRAM, thereby eliminating potential ancestry mismatch effects while ensuring population-specific epistatic interactions were captured. Existing PRS databases predominantly assume additive genetic effects and may not adequately represent the genetic architecture of DM-DR in Korean populations. PRS variants were derived entirely from this Korean cohort through: (1) GWAS analysis of DM-DR vs DM-NR, (2) GMDR analysis to identify epistatic interactions, (3) selection of the top three-way interaction, and (4) cross-validation to prevent overfitting.

Internal Validation and Overfitting Prevention: Ten-fold cross-validation was performed by randomly dividing the dataset into 10 equal subsets, with each fold used once as validation data while the remaining 9 folds served as training data. Model consistency was evaluated by examining variant

selection stability across all folds. To address potential overfitting concerns given the modest DM-DR sample size (n=165), rigorous internal validation was performed using 10-fold cross-validation. The final 3-SNP model achieved perfect cross-validation consistency (10/10), demonstrating robust model stability. Additional models (9-SNP and 10-SNP) were also evaluated to ensure optimal model selection. **Risk Stratification Approach:** The final 3-SNP PRS generated scores ranging from 0 to 6 risk alleles. Given that the majority of samples clustered around scores of 3-4 and our modest DM-DR sample size (n=165), tertile stratification was deemed most appropriate to ensure adequate statistical power while maintaining clinical interpretability. PRS tertiles were defined as: low-risk (0-2 risk alleles, n=3,880), medium-risk (3-4 risk alleles, n=1,082), and high-risk (5-6 risk alleles, n=192). This stratification approach provided balanced group sizes suitable for robust statistical analysis while avoiding over-stratification that could compromise statistical power in smaller subgroups **Performance Evaluation:** Discriminative performance was assessed using the area under the receiver operating characteristic curve (AUC) with 95% confidence intervals by logistic regression models adjusted for both covariate sets. Participants were stratified into three risk categories (low-PRS, medium-PRS, high-PRS) with thresholds determined to ensure adequate group sizes while maintaining biological interpretability [6].

2.8. Multi-marker Analysis of GenoMic Annotation (MAGMA) and Pathway Analysis

We performed gene-level and pathway analyses using MAGMA v1.10. Selected genes associated with genome-wide significant variants from our GWAS were mapped using NCBI build 37 (GRCh37). MAGMA analysis was used to compute gene-level statistics accounting for LD using the 1000 Genomes East Asian reference panel. Pathway enrichment analysis was conducted on the selected gene set using curated gene sets from MSigDB, including KEGG and GO databases. A competitive gene-set analysis framework was applied, and statistical significance was determined using false discovery rate (FDR) correction to control for multiple pathway testing, with significance defined as FDR-adjusted $q < 0.05$.

2.9. Sensitivity analysis based on diabetic duration

To assess the robustness of PRS and DM-DR associations, we conducted sensitivity analyses using three cohorts with progressive diabetic duration restrictions: (1) full cohort without exclusions, (2) diabetes duration ≤20 years, and (3) diabetes duration ≤10 years. Logistic regression models with consistent covariate adjustment were applied to each cohort, calculating odds ratios (OR) and 95% confidence intervals (CI) for PRS categories (low, medium, and high) using low-PRS as a reference. Heterogeneity across cohorts was assessed using Cochran's Q test ($p>0.05$ indicating no significant heterogeneity) and I^2 statistics (<25% indicating low heterogeneity). This approach evaluated whether genetic associations remained stable across different diabetic durations and assessed the potential contributions of genetic risk to the earlier onset of diabetic retinopathy.

References

1. Park, S.; Kang, S. Association between Polygenetic Risk Scores of Low Immunity and Interactions between These Scores and Moderate Fat Intake in a Large Cohort. *Nutrients* **2021**, 13(8).
2. Rabbee, N.; Speed, T.P. A genotype calling algorithm for Affymetrix SNP arrays. *Bioinformatics* **2006**, 22(1), 7-12.
3. Uma Jyothi, K.; Reddy, B. M., Gene-gene and gene-environment interactions in the etiology of type 2 diabetes mellitus in the population of Hyderabad, India. *Meta gene* **2015,** 5, 9-20.
4. Lou, X. Y.; Chen, G. B.; Yan, L.; Ma, J. Z.; Zhu, J.; Elston, R. C., et al., A generalized combinatorial approach for detecting gene-by-gene and gene-by-environment interactions with application to nicotine dependence. *Am J Hum Genet* **2007,** 80, 1125-37.
5. Dudbridge, F., Power and Predictive Accuracy of Polygenic Risk Scores. *PLOS Genetics* **2013,** 9, e1003348.
6. Park, S.; Zhang, X.; Lee, N. R.; Jin, H. S., TRPV1 Gene Polymorphisms Are Associated with Type 2 Diabetes by Their Interaction with Fat Consumption in the Korean Genome Epidemiology Study. J Nutrigenet Nutrigenomics 2016, 9, 47-61.
